# MicroRNA expression as risk biomarker of breast cancer metastasis: a pilot retrospective case-cohort study

**DOI:** 10.1186/1471-2407-14-739

**Published:** 2014-10-02

**Authors:** Augusto LF Marino, Adriane F Evangelista, René AC Vieira, Taciane Macedo, Ligia M Kerr, Lucas Faria Abrahão-Machado, Adhemar Longatto-Filho, Henrique CS Silveira, Marcia MC Marques

**Affiliations:** Molecular Oncology Research Center, Barretos Cancer Hospital, Barretos, 14784-400 Brazil; Department of Mastology and Breast Reconstruction, Barretos Cancer Hospital, Barretos, 14784-400 SP Brazil; Department of Pathology, Barretos Cancer Hospital, CEP: 14784-400 Barretos, SP Brazil; Barretos School of Health Sciences - FACISB, Barretos, São Paulo Brazil; Laboratory of Medical Investigation (LIM) 14, Department of Pathology, University of São Paulo School of Medicine, São Paulo, SP 1246903 Brazil; Life and Health Sciences Research Institute (ICVS), School of Health Sciences, University of Minho, 4704-553 Braga, Portugal; ICVS/3B’s-PT Government Associate Laboratory, 4710-057 Braga/Guimarães, Portugal

**Keywords:** Breast cancer, Metastasis, MicroRNAs, Biomarkers, Molecular profile, Retrospective study

## Abstract

**Background:**

MicroRNAs (miRNAs) are small, non-coding RNA molecules involved in post-transcriptional gene regulation and have recently been shown to play a role in cancer metastasis. In solid tumors, especially breast cancer, alterations in miRNA expression contribute to cancer pathogenesis, including metastasis. Considering the emerging role of miRNAs in metastasis, the identification of predictive markers is necessary to further the understanding of stage-specific breast cancer development. This is a retrospective analysis that aimed to identify molecular biomarkers related to distant breast cancer metastasis development.

**Methods:**

A retrospective case cohort study was performed in 64 breast cancer patients treated during the period from 1998–2001. The case group (n = 29) consisted of patients with a poor prognosis who presented with breast cancer recurrence or metastasis during follow up. The control group (n = 35) consisted of patients with a good prognosis who did not develop breast cancer recurrence or metastasis. These patient groups were stratified according to TNM clinical stage (CS) I, II and III, and the main clinical features of the patients were homogeneous. MicroRNA profiling was performed and biomarkers related to metastatic were identified independent of clinical stage. Finally, a hazard risk analysis of these biomarkers was performed to evaluate their relation to metastatic potential.

**Results:**

MiRNA expression profiling identified several miRNAs that were both specific and shared across all clinical stages (p ≤ 0.05). Among these, we identified miRNAs previously associated with cell motility (let-7 family) and distant metastasis (hsa-miR-21). In addition, hsa-miR-494 and hsa-miR-21 were deregulated in metastatic cases of CSI and CSII. Furthermore, metastatic miRNAs shared across all clinical stages did not present high sensitivity and specificity when compared to specific-CS miRNAs. Between them, hsa-miR-183 was the most significative of CSII, which miRNAs combination for CSII (hsa-miR-494, hsa-miR-183 and hsa-miR-21) was significant and were a more effective risk marker compared to the single miRNAs.

**Conclusions:**

Women with metastatic breast cancer, especially CSII, presented up-regulated levels of miR-183, miR-494 and miR-21, which were associated with a poor prognosis. These miRNAs therefore represent new risk biomarkers of breast cancer metastasis and may be useful for future targeted therapies.

**Electronic supplementary material:**

The online version of this article (doi:10.1186/1471-2407-14-739) contains supplementary material, which is available to authorized users.

## Background

Breast cancer is the most frequent tumor after skin malignancies, representing the second most common cancer-related mortality in women [1]. Although TNM staging provides important clinical prognostic information, mammary tumors are known to be biologically heterogeneous with regard to therapeutic responses as well as molecular profiling [2, 3]. For example, these tumors can be characterized as luminal A, luminal B, basal-like, human epidermal growth factor receptor 2 (HER-2)-overexpressing or claudin-low [4], and this profiling provides additional molecular prognostic markers. In addition, a recent study demonstrated that the microRNA expression signature appears to provide a better characterization of cancer subtypes than transcriptional profiles and may therefore represent a new classification system for breast cancer [5].

MicroRNAs (miRNAs) are small, non-coding RNAs of 19–25 nt that control a wide array of physiological and pathological processes by modulating the expression of their cognate target genes through cleaving mRNA molecules or inhibiting their translation [6]. Most cancer tissues are archived as formalin-fixed, paraffin-embedded (FFPE) samples, and microRNAs are promising biomarkers because they are a stable form of RNA. Moreover, recent studies have shown a good correlation between microRNA samples from frozen and FFPE sections [7, 8].

MiRNAs regulate key biological processes such as development, differentiation, stress response, apoptosis and proliferation [9–11] and are consequently implicated in several diseases including cancer [12–14].

Iorio et al. [13] demonstrated the influence of miRNA deregulation in the development of breast cancer in several tissues and lineages, and other studies have correlated miRNA profiles with mRNA subtypes, particularly with regard to estrogen receptor (ER), progesterone receptor (PR) and HER2 status [4, 14]. In addition, specific miRNAs have been associated with steps of the metastasis cascade, such as micrometastasis, local invasion, intravasation and metastatic colonization [15]. However, in practice, few miRNA expression signatures have been shown to correlate with breast cancer metastasis, and although the epithelial to mesenchymal transition (EMT) is a critical event for metastasis from carcinomas, few studies evaluating miRNAs during EMT have been performed in breast cancer [16, 17]. In breast cancer, one example is miR-183, which have been associated with migration and invasion [18, 19]. However, the results regard to miR-183 is controversial. In a recent study of meta-analysis, comparing cancer tissues with controls of several tumor types, this miRNA presented inconsistent regulation in breast cancer [20]. However, miR-183 expression has not been evaluated in a metastatic breast cancer context.

The main goal of this study was to identify miRNA biomarkers of breast cancer metastasis. Using a collection of FFPE samples, we selected clinically homogeneous samples, and we paired metastatic and non-metastatic patients according to tumor grade. Using this strategy, we detected, with improved precision, miRNA biomarkers that could characterize metastasis irrespective of clinical staging as well as stage-specific biomarkers.

## Methods

### Study population

A retrospective case cohort study [21] was performed in 782 patients with invasive breast cancer (ductal or lobular), without metastasis at diagnosis (clinical stage I, II and III) who had previously received treatment at Barretos Cancer Hospital between 1998 and 2001. In this study, the case group consisted of patients with a poor prognosis who developed breast cancer recurrence and/or metastasis during a follow up of ten years. The rate of cases with metastasis according each clinical stage was: 12.8% to CSI (total of 117), 25% to CSII (total of 352), 51% to CSIII (total of 313).

The control group consisted of a random sample of patients who did not develop breast cancer recurrence and/or metastasis and had a good prognosis. The control group presented the same apparent risk and length of follow up period as the case group. The rate of cases with non-metastasis according each clinical stage was: 87.2% to CSI (117 total cases), 75% to CSII (total cases 352), 49% (313 total cases).

The groups also had a similar distribution of clinical staging (CS) according to TNM classification (TNM 7th edition) [2]. All patients were treated at the same institution with same treatment protocol and received regular follow-up assessments at the Department of Mastology and Reconstructive Surgery at Barretos Cancer Hospital, Barretos, Sao Paulo, Brazil. Patients were excluded if they had a second primary tumor, an insufficient blocked tumor or the absence of high-quality miRNA for extraction. This study was approved by Barretos Cancer Hospital ethical committee, (protocol n°362/2010).

### Pathologic evaluation

The same pathologist reviewed all of the medical records. Immunohistochemistry evaluation was performed in all cases, including the assessment of ER and PR status and the expression of Ki-67, Her2 and cytokeratin 5/6. ER status was evaluated using the Pathway anti-Her-2 790–2991 monoclonal antibody (Ventana Medical Systems, Roche Diagnosis, Tucson, Arizona 85755, USA) at a dilution of 1:200.

PR status was evaluated using the Rabbit monoclonal antibody clone clone SP1 at a dilution of 1:600. Ki-67 expression was evaluated using the monoclonal antibody MIB-1 (Dako, Sao Paulo, Brazil) at a dilution of 1:200. Her2 status was evaluated using the mouse antibody clone 4B5 (Ventana Medical Systems, Roche Diagnosis, Tucson, Arizona 85755, USA) at a 1:2,000 dilution.

The cytokeratin 5/6 status was evaluated using the mouse monoclonal D5/16B4 (Dako, Sao Paulo, Brazil) at a 1:100 dilution. ER and PR expression was considered positive when 1% of tumor cells showed positive staining. The Ki-67 cutoff value was 14%. For Her2 semi-quantitative immunohistochemistry (2+ and 3+), the DISH test was performed using Her2 Dako K5331 (Dako, Sao Paulo, Brazil). Basal-like tumors were characterized according to triple-negative receptor status and positive C5/6 status. We applied immunohistochemistry molecular characteristic subgroups based on previous reports [22, 23].

### Patients and case selection

During the paring we considered a case-cohort ratio 1:1. After using the inclusion and exclusion criteria, 64 patients were selected including 29 in the case group and 35 in the control group. Ductal invasive carcinoma represented 76.6% of the group histology. The average patient age was 53.1 years (29–95), and the average tumor size was 3.1 cm (1.0-8.5 cm).

The groups were homogeneous, and there were no differences between the variables selected prior to pairing. Table 1 summarizes the main patient features, with the associated p-values from the Fisher test using SPSS software. Using a t-test for independent variables, there were no differences between groups related to tumor size (mean 3.24 × 3.04; p = 0.592) or age (mean 49.1 × 56.4; p = 0.07). After pairing and pathologic classification, we observed that only the PR status differed between the groups (Table 1).

**Table 1:** **Clinical and pathological features of the patient groups according to Fisher’s test**

Recurrence		Absent	Present	Total	p value
**Selected variables before pairing**					
CS-TNM	EC I	9 (69%)	4 (31%)	13	0.385
	EC II	13 (56%)	10 (43%)	23	
	EC III	13 (46%)	15 (54%)	28	
CS-T (TNM)	T1	15 (56%)	12 (44%)	27	0.742
	T2	10 (56%)	8 (44%)	18	
	T3	6 (67%)	3 (33%)	9	
	T4	4 (40%)	6 (60%)	10	
CS-N (TNM)	N0	17 (68%)	8 (32%)	25	0.378
	N1	9 (47%)	10 (53%)	19	
	N2	5 (50%)	5 (50%)	10	
	N3	4 (40%)	6 (60%)	10	
**Categorical variables observed after pairing**					
Histology	Ductal	30 (61%)	19 (39%)	49	0.078
	Lobular	5 (33%)	10 (67%)	15	
ER	ER +	21 (62%)	13 (38%)	34	0.226
	ER -	14 (47%)	16 (53%)	30	
PR	PR +	16 (76%)	5 (24%)	21	0.018
	PR -	19 (44%)	24 (56%)	43	
Her2	Her2 +	5 (38%)	8 (62%)	13	0.158
	Her2 -	30 (59%)	21 (41%)	51	
Molecular	Luminal	23 (64%)	13 (36%)	36	0.265
Subtypes	Basal like	9 (45%)	11 (55%)	20	
	Her2	3 (38%)	5 (62%)	8	

ER = estrogen receptor; PR = progesterone receptor.

The mean group follow-up duration was 82.0 months (5.1-162 months); excluding the case group, this period was 120.6 months. At the end of the study, 25/29 patients in the case group had died of cancer, while the others remained alive with tumor recurrence.

### Total RNA isolation from FFPE sections

FFPE samples were submitted to a total RNA isolation protocol using the Recover All ^TM^ Total Nucleic Acid Isolation kit (Life Technologies). The samples were initially treated with xylene, followed by double washing with absolute ethanol and proteinase K treatment at 50°C for 3 hours. Quantification was performed using a nanodrop ND-1000 spectrophotometer (NanoDrop Products, Wilmington, DE), and RNA quality was assessed using an Agilent Small RNA chip with a Bioanalyzer device (Agilent Technologies).

### miRNA microarrays

The Agilent Human miRNA Microarray (8 × 15K - G4471A, Agilent Technologies) was used in all samples from FFPE sections. Additional microarrays of frozen samples were performed in comparison to those obtained from FFPE, as quality control (Additional file 1: Figure S2). A total of 100 ng of total RNA was hybridized using miRNA complete labeling and the Hyb Kit (Agilent Technologies), according to the manufacturer’s instructions. The reactions followed a 2-step preparation, represented by dephosphorylation and denaturation of the total RNA incorporated with Cy3 fluorochrome by the T4 ligase. The next steps included standard washing procedures and hybridization with microarrays slides. The images were scanned using an Agilent DNA microarray scanner with SureScan technology (Agilent Technologies).

### miRNA microarray data analysis

The raw data were obtained using Feature Extraction software v.11.0 (Agilent Technologies) and submitted to R environment v. 2.15.0 [24] for further analysis. The median signals (*gMedianSignal* and *gBGMedianSignal*) were used. Following background subtraction and log2 scale transformation, normalization was performed using the quantile method with the aroma light package [25]. Differentially expressed microRNAs were obtained by rank product analysis using the RankProd package [26], considering p-values and positive false predictions (pfp) ≤ 0.05. The rank product analyses were separated according to clinical staging between groups (CSI-CSIM, CSII-CSIIM, CSIII-CSIIIM). Differentially expressed miRNAs were further ranked according to sensitivity and specificity to determine the best candidates between non-metastatic patients and metastatic patients in a stage-specific manner. Sensitivity and specificity are defined as the number of true positive decisions/the number of actually positive cases and the number of true negative decisions/the number of actually negative cases. The area under the ROC curve (AUC) is a measure for overall performance, which can be interpreted as the average value of sensitivity for all possible values of specificity [27]. The criteria for biomarker selection included sensitivity and specificity values ≥ 80%, as determined using the ROCR package [28]. Differentially expressed microRNAs were clustered by Euclidian distance and average linkage using the heatmap.2 function of the gplots package [29].

### Real-time PCR and disease-free survival analysis

The criteria for miRNA selection for further RT-qPCR confirmation, and further analysis, was the high significance (pvalue and pfp) of the miRNA, biological relevance after literature search and which is present in (1) both groups and (2) at least 1 miRNA specific of each metastatic group compared to their specific primary patient of the same CS. We performed a Venn diagrams’ to represent this selection, using gplots package [29].

Taqman microRNA assays (Life Technologies, Foster City, CA, USA) were used to confirm the microarray data. In brief, these reactions consist of reverse transcription with miRNA-specific primers in a real-time PCR reaction with Taqman probes. The reverse transcriptase reactions used in this study contained 10 ng of total RNA and utilized the High Capacity cDNA Reverse Transcription Kit (Life Technologies), according to the manufacturer’s instructions, in a thermocycler (Eppendorf). All real-time PCR reactions were performed in triplicate in a 7900 HT Fast Real-time PCR System (Applied Biosystems USA). All analysis procedures were performed in R environment. The normalization step was performed according to the 2^-ΔΔCt^ method [30]. Cycle threshold (Ct) values from selected miRNA targets were subtracted from the Ct values of the endogenous small noncoding RNA control RNU48 (Control miRNA Assay, *Applied Biosystems*, Foster City, CA, USA). A subsequent ΔΔCt value was calculated by subtracting metastatic ΔCt values from non-metastatic ones. The data cutoff for modulation (up/down) in each patient was estimated according to the threshold obtained after receiver operating characteristic (ROC) curve analysis using the ROCR package. To assess the combination of biomarkers, a general logistic model (glm) was performed prior to ROC analysis. MiRNA modulation (up/down) was used, and data from the first clinical evaluation until recurrence were considered for the non-parametric estimation of disease-free survival using the Kaplan-Meier method. Risk curves were used, and comparisons were performed using the log-rank test and the Cox hazard model, considering p-values ≤0.05. In multiple Cox analysis, each clinical variable, such as molecular subtype, TNM and histology (ductal and lobular), was compared separately with the miRNA expression. The survival R package was used in this analysis [31].

### Tissue microarray analysis

Considering that the three selected miRNAs shared PTEN as target we performed a tissue microarray analysis of these 64 cases of breast cancer, in duplicate, to evaluate the expression of this protein in both group of patients (metastatic and metastatic breast cancer). The TMAs were assembled using a manual tissue microarrayer (Beecher Instruments, Silver Springs, MD - USA). The TMA was done, with control tissues (testis and placenta tissue) and a series of 64 duplicate samples of breast ductal carcinoma and lobular histology were related to clinical stage I, II and III the same samples used in the screening by microarray, totaling 128 points in the receptor block.

Sections of 5 μm were cut from the TMA and further processed to immunostaning with PTEN antibody. For the immunohistochemistry of PTEN a Cell Signaling Monoclonal (reference number 9551P) antibody was used, diluted 1:250. Deparaffinised formalin-fixed, paraffin-embedded tissue sections were heated in the pretreatment module of the autostainer in Tris–HCl pH 8.5 buffer (for 20 minutes at 98°C). To detect the immune reaction we used the sequential system with peroxidase-conjugated secondary antibody (Abcam, USA) or the amplification system SS polymer Polymer Volume Mega-HRP Detection Kit (BioGenex, USA) according to the manufacturer's specifications. For the development of the tags the chromogen diaminobenzidine, DAB (Sigma, USA) and counter-stained with hematoxylin was used. Images of histological sections were digitized using Eclipse 50i microscope (Nikon, Japan) coupled to a digital video camera Sight DS system - Fi1 (Nikon, Japan) with an image analyzer Image-Pro Express version 6.0 (Media Cybernetics, USA).

### Functional analysis

Target prediction was performed using the mirDIP interface [32]. In the present search, we selected at least 3 of 12 algorithms available for prediction. The targets were analyzed by functional analysis using the Database for Annotation, Visualization and Integrated Discovery (DAVID) version 6.7 [33, 34]. This approach was used to identify significant biological processes and pathways that could be shared between targets of miRNAs of interest. A biological process or pathway was considered significant if it contained a minimum of 3 genes per category, featuring score values less than 0.05 including the Benjamini-Hochberg correction.

## Results

The present study included 64 breast cancer patients, including non-metastatic patients (n = 35) and those with metastatic recurrence or metastasis at follow up (n = 29). The groups were stratified according to clinical staging (CS) as CSI, CSII and CSIII with the intention of identifying specific and shared miRNAs associated with metastasis. There were no differences between the groups before pairing with regard to CS; after pairing, the only difference between the groups was related to PR status (p = 0.02; Table 1).

### miRNAs differentially expressed between non-metastatic and metastatic patients

**Figure 1:**
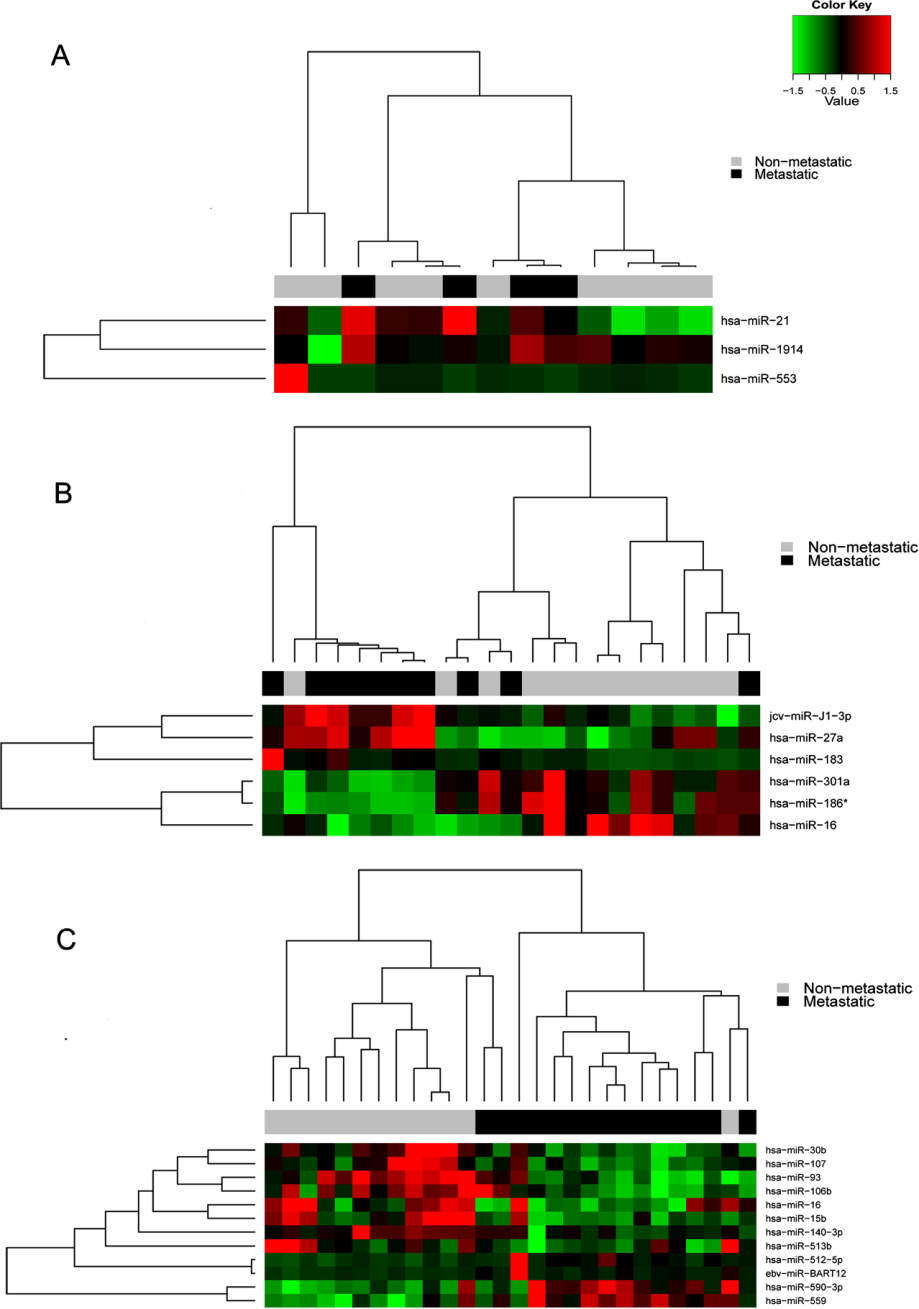
**Heatmaps of the best biomarkers stratified according to clinical stage.** Figure 1 **A** shows non-metastatic vs. metastatic patients in CSI, Figure 1 **B** shows patients in CSII, and Figure 1 **C** shows patients in CSIII.

The differentially expressed miRNAs (non-metastatic vs. metastatic groups) stratified according to clinical stage (CSI, CSII and CSIII) are shown in Additional file 2: Figure S1. The optimal biomarkers were ranked according to sensitivity and specificity values ≥ 80%. The clusters resulting from this analysis are represented in Figure 1, revealing miRNAs highly specific for the metastatic process. The miRNA hsa-miR-183 (CSII) was also selected for further analysis.

**Figure 2:**
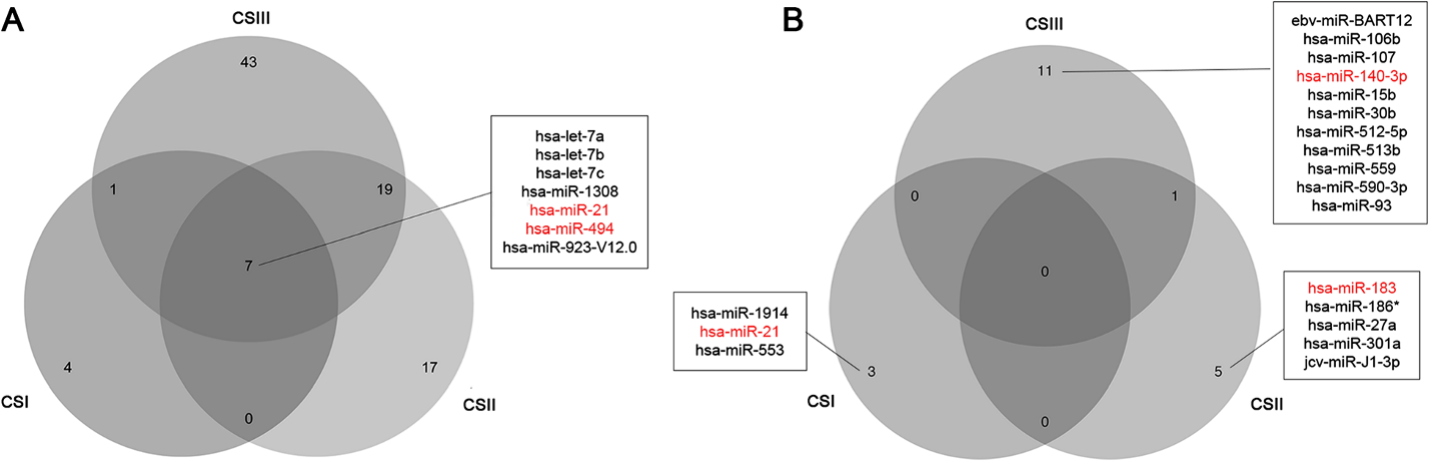
**Venn diagram showing the differentially expressed genes after rank product analysis between CSI, CSII and CSIII non-metastatic and metastatic paired groups.** Figure 2 **A** shows the 7 genes shared between all analyses are represented and Figure 2 **B** shows the best biomarkers according to sensitivity and specificity values (≥ 80%). The miRNAs selected for real-time PCR confirmation are highlighted in red.

Seven microRNAs were identified in the metastatic group irrespective of clinical staging (hsa-let-7a, hsa-let-7b, hsa-let-7c, hsa-miR-1308, hsa-miR-21, hsa-miR-494 and hsa-miR-923_v12.0, Figure 2). Of these, hsa-miR-494 and hsa-miR-21 were selected for further analysis using real-time PCR.

### Relative risk analysis

The miRNA expression levels of the 64 patients evaluated in the microarray were assessed using real-time PCR for relative risk analysis. The primers used are shown in Additional file 3: Table S1.

The 3 microRNAs selected for further real-time PCR analysis shared important biological features, especially with regard to phosphoprotein- and kinase protein-associated functions, according to functional analysis.

The ROC curve, considering the non-metastatic group as the reference, was used to select the threshold value for miRNA modulation of quantitative values obtained by real-time PCR. The values for the area under the curve (AUC), sensitivity and specificity are presented in Table 2.

**Table 2:** **ROC curve analysis for real-time PCR threshold value determination of miRNAs in CSII patients**

microRNA	Threshold value	AUC	Sensitivity (%)	Specificity (%)
miR-21	1.89	0.438	30	75
miR-183	4.708	0.769	75	80
miR-494	1.688	0.400	30	75
miR-21 + miR-494	0.497	0.600	50	75
miR-21 + miR-183	0.431	0.823	90	70
miR-183 + miR-494	0.354	0.830	70	75
miR-183 + miR-21 + miR-494	0.567	0.838	75	80

**Figure 3:**
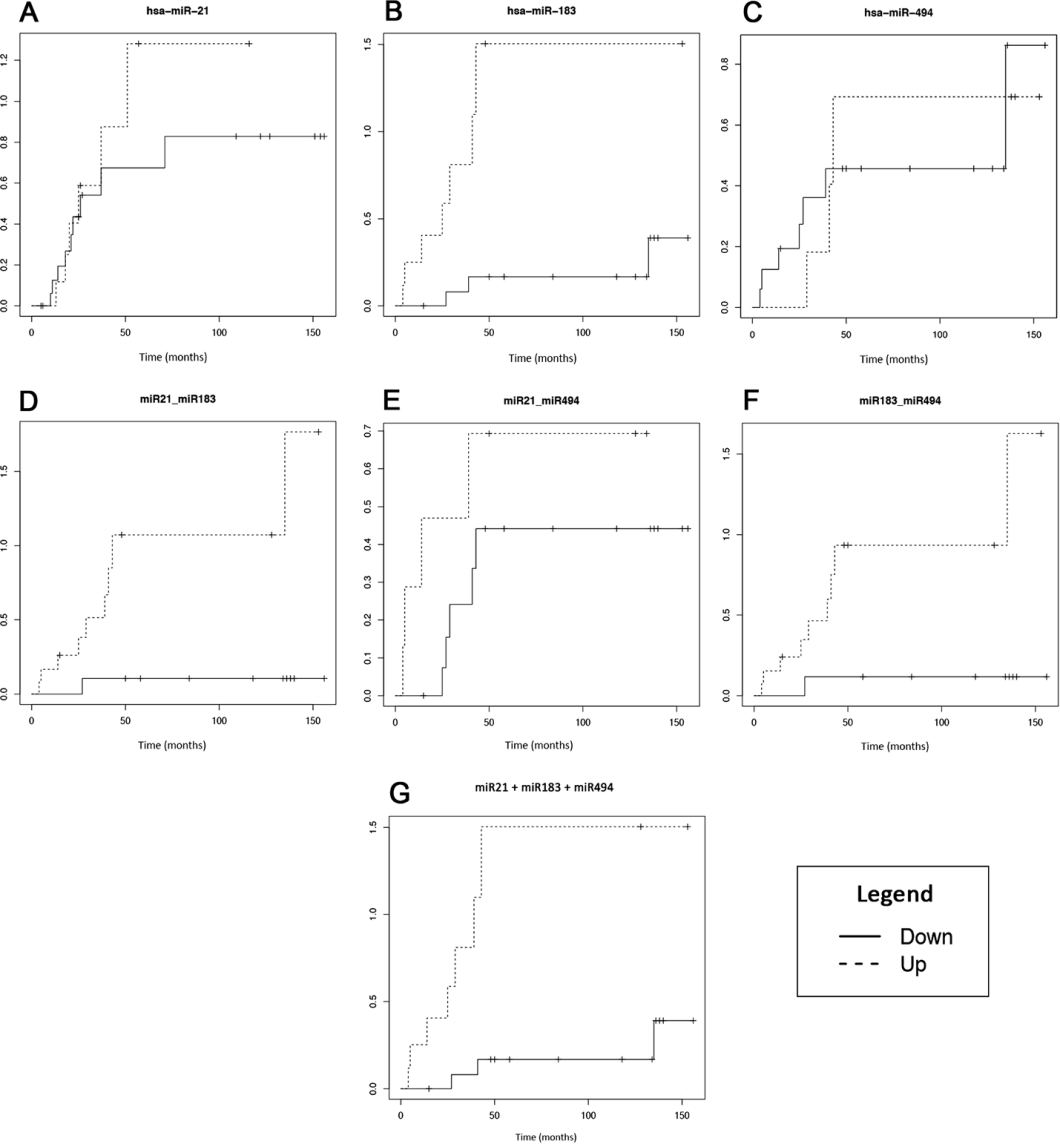
**Relative risk curves of the best biomarkers in CSII patients.** In **A**, **B** and **C**, miRNAs miR-21, miR-183 and miR-494 were analyzed separately. In **D**, the combination of miR-21 and miR-183 was analyzed. In **E**, the combination of miR-21 and miR-494 was analyzed. In **F**, the miR-183 and miR-494 combination was analyzed. In **G**, the combination of the three miRNAs studied (miR-21, miR-183 and miR-494). The miRNA up-regulation is represent by dotted lines, and down-regulation is represented by a continuous line.

The relative risk curves using recurrence data as events are shown in Figure 3. Because of the small number of CSI patients, it was not possible to perform this analysis for this group. For CSII patients, hsa-miR-183 was the only miRNA that was significant when analyzed independently using the log-rank test (p = 0.03). In contrast, hsa-miR-21 and hsa-miR-494 showed no significance (p = 0.88 and 0.86, respectively). After combining the up-regulated levels of these 3 miRNAs our analysis, showed that were associated with metastatic events (p = 0.002). The combination of hsa-miR-21 with hsa-miR-494 was not significant after the log-rank test (p = 0.123), whereas other combinations such as hsa-miR-21 with hsa-miR-183 (p = 0.004) and hsa-miR-183 with hsa-miR-494 (p = 0.001) were significant. Among CSIII cases, we did not find significant results (data not shown). The miRNA expression and clinical variable data were included in the multiple Cox models, and the best results are shown in Table 3.

**Table 3:** **Cox hazard models used in combination with hsa-miR-21, hsa-miR-494 and hsa-miR-183 in CSII patients**

Covariate	Category	HR (95% CI)	p value
Histology	Ductal	1	0.1105
	Lobular	3.374 (0.7577-15.02)	
miRNA regulation	Down	1	0.0134
	Up	5.8382 (1.44192-23.64)	
T-TNM	Other	1	0.41401
	T2	1.7449 (0.4589-6.635)	
miRNA regulation	Down	1	0.00604
	Up	7.584 (1.7856-32.207)	
N-TNM	N0	1	0.30875
	N1	2.009 (0.5244-7.695)	
miRNA regulation	Down	1	0.00733
	Up	6.6417 (1.664-26.503)	
Molecular subtype	Other	1	0.08739
	Her2	3.766 (0.8233-17.23)	
miRNA regulation	Down	1	0.00528
	Up	8.332 (1.8785-36.95)	

The main characteristics that provided some degree of risk included lobular histology, T2, N1 and the Her2 molecular subtype. However, none of these factors were significant in CSII patients and miRNA expression was a more effective independent prognostic factor in all cases.

**Figure 4:**
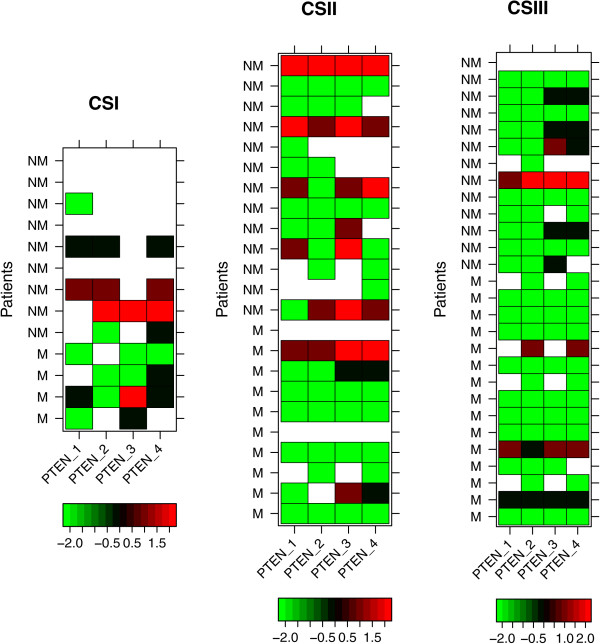
**Heat maps of PTEN hybridizations by tissue microarrays (TMA) for CSI, CSII and CSIII patients.** Each row represents the staining level for the proteins in non-metastatic (NM) or metastatic (M) patients. Each column shows PTEN hybridizations performed in duplicates, analyzed by two different pathologists (showing four results). The color key scale represented by bright red (level 2) indicates score 3, weak red (level 1) for score 2, black (level 0) for score 1 and green (level -2) for no staining (score = 0). Missing data is represented in white.

Finally, our results showed that PTEN protein could not be detected in almost all cases of breast cancer samples when the three miRNAs were induced by TMA methodology (Figure 4).

## Discussion

Despite recent findings regarding the role of microRNAs in metastasis, the molecular mechanisms of breast cancer progression remain incompletely understood. For example, the prognostic significance of tumor grade in this type of cancer remains unknown, as well as the molecular mechanisms for why small tumors from CSI (as well as those from CSII and CSIII) can lead to metastasis, which presented 10-years of follow-up. The CSI metastatic samples are extremely rare to obtain, and, in the present study it was possible to obtain four cases in hundreds of patients analyzed. Although there were difficulties associated with obtaining sufficient numbers of CSI metastatic samples, the similarities between tumors of different clinical stages were considered. We performed a retrospective study and identified new biomarkers of breast cancer metastasis that are both shared and specific to clinical stages I-III using FFPE samples. Despite low quality of FFPE samples, several studies have showed the stability of miRNAs [7, 8], because may be less affected than mRNA by formalin fixation and paraffin embedding perhaps due to their smaller size and lack of poly A tails [35]. In the present study, an additional quality control was performed, showing high correlation of frozen samples and FFPE samples used (Additional file 1: Figure S2).

The first application of microarray-based gene expression profiling analysis to the study of breast cancer consisted of disease assessment at the molecular level. In addition, class-prediction studies aim to identify miRNA predictors that could be applicable to all patients with breast cancer, with the goal of separating patients according to prognosis and selecting candidate genes for metastasis during follow up [36].

The Mamaprint, Oncotype DX and Breast Cancer Index selected patients who were ER positive, and the Veridez 76-gene study evaluated patients without lymph node metastases [37]. The present case cohort study evaluated patients with invasive breast cancer based on metastasis development and selected microRNAs related to metastasis development independent of lymph node or hormonal status.

The Oncotype DX study initially evaluated a retrospective cohort, and the genes of interest were selected using a univariate Cox analysis with a median of 15.1 years of follow up [38]. The Mamaprint study evaluated the odds of developing distant metastases after a 5-year follow-up period [39]. We performed a case cohort study with a control group that had a median follow-up duration of 10 years, which decreased the bias related to patient class migration.

The main prognostic factors related to breast cancer are summarized according to the TNM classification; therefore, at diagnosis, the tumor size, lymph node status and distant metastases represent the main prognostic factors. Another independent prognostic factor is the gene signature; however, this is not easy to evaluate in clinical practice. Although a semi-quantitative assessment of ER, PR, HER2 and Ki67 status-using immunohistochemistry is frequently used in clinical practice, this method does not provide the true gene signature [39].

In our study, we standardized the cases and controls according to TNM classification that is considered the main breast cancer prognostic factor and we did not observe any differences between the groups (Table 1). Other variables included in the analysis were related to histology and immunohistochemistry markers. Although the PR status was different between the groups, this did not serve as a prognostic factor, which may be due to the limited number of patients evaluated or a possible bias associated with the group selection.

Considering recent findings regarding breast cancer metastasis, Valastyan [15] reviewed the role of microRNAs according to the steps of the metastatic cascade. In our study, miRNAs shared by clinical stages I-III were found in concordance with processes such as cell motility (let-7 family) and distant metastases (hsa-miR-21). Of these miRNAs, we chose to confirm hsa-miR-21 using real-time PCR because it is the most frequently reported miRNA in several types of solid cancers, representing a potential oncomir [40, 41].

These miRNAs have been described as regulating oncogenes or tumor suppressor genes. The let-7 family appears to regulate the expression of *RAS* and *HMGA2* in breast cancer cells and is associated with several mechanisms of carcinogenesis, including EMT [42, 43]. The other microRNA differentially expressed in both clinical staging, hsa-miR-494, targets several molecules relevant to cancer, including *PTEN* [44]. Transfection studies have shown this miRNA to act as a master cell cycle regulator at the G1/S checkpoint by targeting *CDK6* [45] and at G2/M arrest by targeting *PLK1*, *PTTG1*, *CCNB1*, *CDC2*, *CDC20* and *TOP2A* [46], and this miRNA also affects cell proliferation in A549 lung cells by regulating *IGFBP1* and *IGF2* [47] and in gastrointestinal tumor cells by direct targeting *KIT* [48]. In addition, miR-494 also appears to have a role in TRAIL-induced apoptosis [49] as well as the immune system via its regulation of key transcription factors, such as interferon ɣ and TGFβ1 [50]. The evidence of its role in metastasis was demonstrated as an exossomal miRNA in pre-metastatic sites targeting cadherin-17 in mice [51]. Together, these data as well as the findings of the present study highlight this miRNA as a new interesting candidate for verification in metastatic breast cancer.

Considering the evidences of the importance of miR-21 and miR-494, both confirmed by RT-qPCR in all CS. However, it was not clear the sensitivity and specificity of these biomarkers. After ROC curve analysis, despite the miR-21 was found as differentially expressed in all CS, only presented AUC ≥ 80% in CSI-CSIM and miR-494 were not significative. For these reasons, we also selected other miRNAs of CSII and CSIII to improve the sensibility and specificity.

Supervised analysis, based on clinical staging stratification, identified hsa-miR-183 as the best microRNA with regard to sensitivity and specificity in CSII (Figure 2). The hsa-miR-140-3p, the miRNA tested for CSIII, did not confirm microarray results (data not shown). Of these stage-specific microRNAs, miR-183, which was specific to CSII, is considered an oncogene because it targets *DKK3*, *SMAD4*, *EGR1*, *PTEN* and the PI3K pathway, and it is frequently described in breast cancer as well as other reproductive system related-cancers such as prostate, ovarian and urothelial carcinomas [52, 53]. Moreover, miR-183 has been considered a metastatic inhibitor by targeting ezrin [53] and lymph node metastasis in medullary thyroid carcinoma [54], and this miRNA was recently described as being involved in breast cancer progression [55]. Despite these findings, our study is the first to report the association between miR-183 and CSII patients.

Despite the fact that microRNAs hsa-miR-494 and hsa-miR-21 share important targets such as *PTEN*, this combination was not significant after the log-rank test for CSII or all clinical stages pooled together (data not shown). These miRNAs presented an increased risk only when they were analyzed in combination with miR-183. Moreover, in regards to the molecular mechanisms shared by targets of these miRNAs, we identified phosphoproteins, specifically kinases, among both miRNA targets. For example, *KIT* and *BCL6* were among the hsa-miR-494 proto-oncogenic targets, which represented a significant category with 34 genes, whereas tumor suppressors and apoptotic genes were more evident among hsa-miR-183 target genes, including *PTEN*, *PDCD4* and *BCL10*, as well as tyrosine kinase signaling pathways. Protein expression of PTEN by TMA analysis showed no differences between metastatic and non metastatic breast tumors (Figure 4). The results showed that expression of PTEN protein was repressed in all cases of breast cancer while the three microRNAs are induced. Considering that some spots of TMA was lost, specially to CSI non metastatic tumor samples, probably this could be a limitation to see differences between metastatic group versus non metastatic group about the this protein expression. In our concern further studies using functional assays can be design to better explain the role of these microRNAs in PTEN regulation.

The 3 miRNAs selected (hsa-miR-21, hsa-miR-494 and hsa-miR-183) share *PTEN* as a target, and this combination demonstrated an increased risk for metastasis (Figure 3), which suggests some potentially shared mechanism of action. Moreover, the Cox regression analysis showed that the risk of breast cancer metastasis was more likely to be related to miRNA expression and appeared to be independent of clinic pathological variables. In this study, a homogeneous population was intentionally selected to evaluate the effect of miRNA deregulation with increased efficacy. However, it will be necessary to perform further studies in larger populations to validate these findings. Together, our findings indicate that miRNAs can be independently associated with patient prognosis in breast cancer and may represent risk biomarkers for the development of breast cancer metastasis.

Further studies are necessary to understand the role of these new candidate risk biomarkers and the effects of the combination of these miRNAs in breast cancer metastasis, especially in CSII.

## Conclusions

Taken together, we demonstrate that miR-183, miR-494 and miR-21 were up-regulated in metastatic breast cancer tissues that was associated a poor prognosis. The TMA analysis showed that the expression of PTEN protein was repressed in all cases of breast cancer while the three miRNAs are induced. These data can indicate that these miRNAs represent new risk biomarkers of metastatic breast cancer and may be useful for future targeted studies.

## Electronic supplementary material

Additional file 1: Figure S2: Scatterplot comparing microRNA expression profile of FFPE sections (y-axis) against frozen tissue (x-axis). The *R square* is 0.781. (PDF 135 KB)

Additional file 2: Figure S1: Heatmaps of all differentially expressed miRNAs stratified according to clinical stage. Figure S1A shows non-metastatic vs. metastatic patients in CSI; Figure S1B shows patients in CSII, and Figure S1C shows patients in CSIII. (TIFF 1 MB)

Additional file 3: Table S1: Primers used for real-time PCR confirmation. (DOCX 64 KB)
