# Supplementary figures and images for: MicroRNA expression as risk biomarker of breast cancer metastasis: a pilot retrospective case-cohort study
Source: BMC Cancer. 2014 Oct 2;14:739. doi: 10.1186/1471-2407-14-739 (PMC4195914; doi:10.1186/1471-2407-14-739)

log2 FFPE miRNA

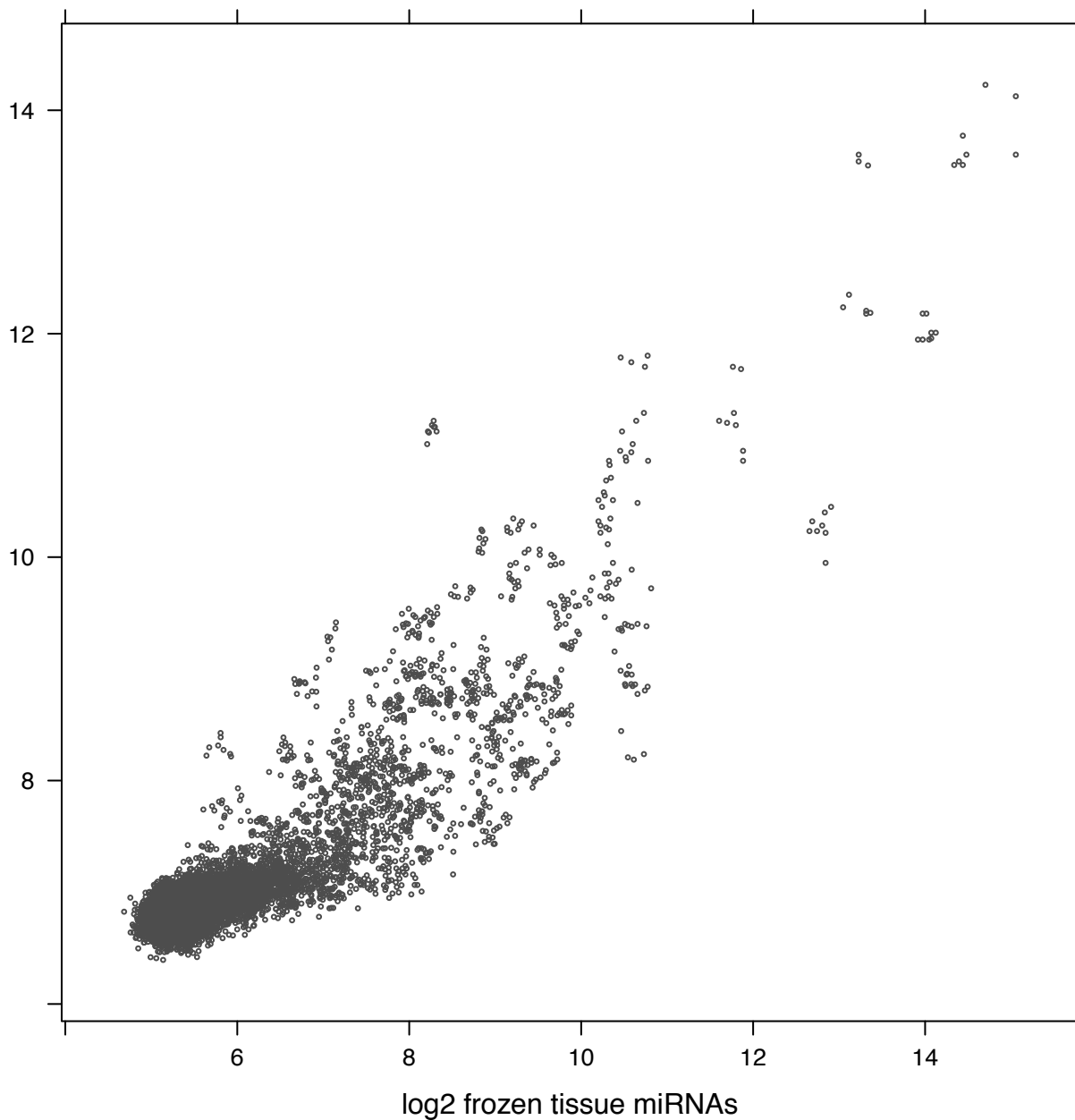

Supplement: Supplementary file 1 — Additional file 1: Figure S2: Scatterplot comparing microRNA expression profile of FFPE sections (y-axis) against frozen tissue (x-axis). The R square is 0.781. (PDF 135 KB) [file 12885_2013_4924_MOESM1_ESM.pdf]

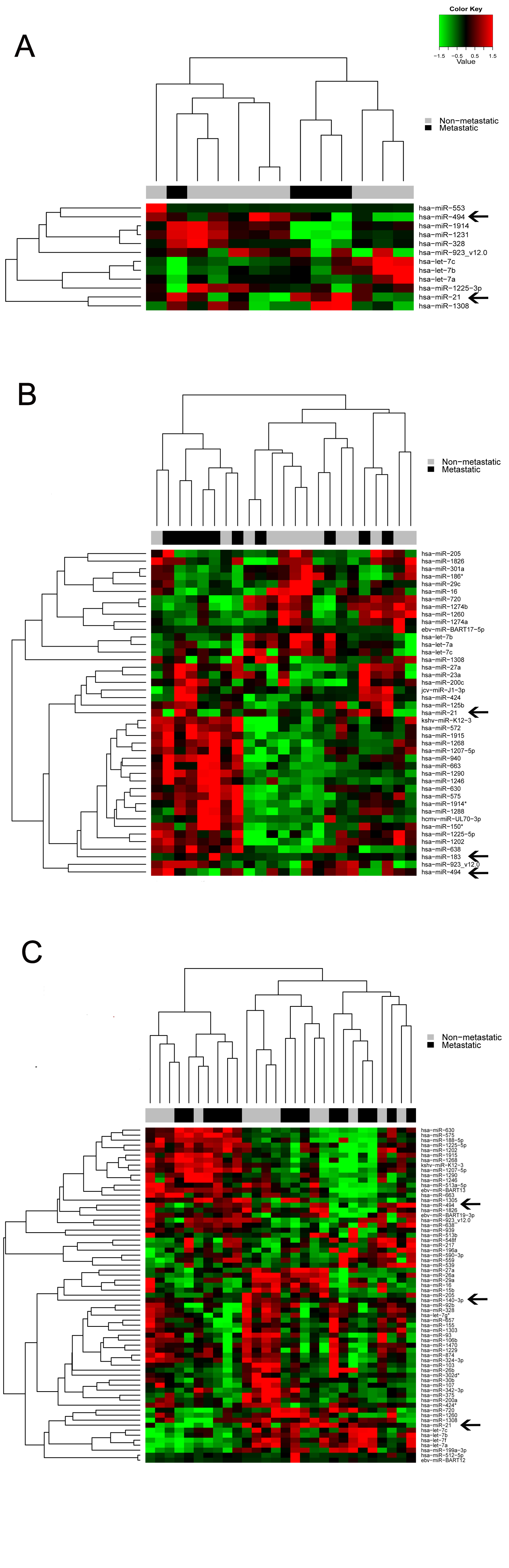

Supplement: Supplementary file 2 — Additional file 2: Figure S1: Heatmaps of all differentially expressed miRNAs stratified according to clinical stage. Figure S1A shows non-metastatic vs. metastatic patients in CSI; Figure S1B shows patients in CSII, and Figure S1C shows patients in CSIII. (TIFF 1 MB) [file 12885_2013_4924_MOESM2_ESM.tiff]
