# Supplementary material for: MicroRNA expression as risk biomarker of breast cancer metastasis: a pilot retrospective case-cohort study
Source: BMC Cancer. 2014 Oct 2;14:739. doi: 10.1186/1471-2407-14-739 (PMC4195914; doi:10.1186/1471-2407-14-739)
Supplement: Supplementary file 3 — Additional file 3: Table S1: Primers used for real-time PCR confirmation. (DOCX 64 KB) [file 12885_2013_4924_MOESM3_ESM.docx]

| microRNA | Accession number | Sequence |
| --- | --- | --- |
| RNU 48 | 001006 | GATGACCCCAGGTAACTCTGAGTGTGTCGCTGATGCCATCACCGCAGCGCTCTGACC |
| hsa-miRNA-183 | 002269 | UAUGGCACUGGUAGAAUUCACU |
| hsa-miRNA-494 | 002365 | UGAAACAUACACGGGAAACCUC |
| hsa-miRNA-21 | 000397 | UAGCUUAUCAGACUGAUGUUGA |
